# Whole exome screening of neurodevelopmental regression disorders in a cohort of Egyptian patients

**DOI:** 10.1007/s10048-022-00703-7

**Published:** 2022-11-26

**Authors:** Miral M. Refeat, Walaa El Naggar, Mostafa M. El Saied, Ayman Kilany

**Affiliations:** 1 Department of Medical Molecular Genetics, Human Genetics and Genome Research Institute, National Research Centre, Cairo, Egypt; 2 Faculty of Medicine, Department of Pediatrics, Cairo University, Giza, Egypt; 3 Department of Research On Children With Special Needs, Medical Research Institute, National Research Centre, Cairo, Egypt

**Keywords:** Developmental regression, Neurodegenerative diseases, Leukodystrophy, Neuronal ceroid lipofuscinosis diseases, Whole exome sequencing

## Abstract

Developmental regression describes a child who begins to lose his previously acquired milestones skills after he has reached a certain developmental stage and though affects his childhood development. It is associated with neurodegenerative diseases including leukodystrophy and neuronal ceroid lipofuscinosis diseases (NCLs), one of the most frequent childhood-onset neurodegenerative disorders. The current study focused on screening causative genes of developmental regression diseases comprising neurodegenerative disorders in Egyptian patients using next-generation sequencing (NGS)-based analyses as well as developing checklist to support clinicians who are not familiar with these diseases. A total of 763 Egyptian children (1 to 11 years), mainly diagnosed with developmental regression, seizures, or visual impairment, were studied using whole exome sequencing (WES). Among 763 Egyptian children, 726 cases were early clinically and molecularly diagnosed, including 482 cases that had pediatric stroke, congenital infection, and hepatic encephalopathy; meanwhile, 192 had clearly dysmorphic features, 31 showed central nervous system (CNS) malformation, 17 were diagnosed by leukodystrophy, 2 had ataxia telangiectasia, and 2 were diagnosed with tuberous sclerosis. The remained 37 out of 763 candidates were suspected with NCLs symptoms; however, 28 were confirmed to be NCLs patients, 1 was Kaya-Barakat-Masson syndrome, 1 was diagnosed as infantile neuroaxonal dystrophy, and 7 cases required further molecular diagnosis. This study provided an NGS-based approach of the genetic causes of developmental regression and neurodegenerative diseases as it comprised different variants and de novo mutations with complex phenotypes of these diseases which in turn help in early diagnoses and counseling for affected families.

## Introduction

Developmental regression is a complex phenomenon that has been described in different developmental disorders and defined as the early absence of acquired milestones skills which affects the brain and behavioral development [1]. It differs from developmental delay that children suffering developmental delay do not achieve developmental milestones in comparison to those of the same age range, while a child experiencing developmental regression refers to a normal developmental phase, followed by loss of previously acquired skills [2]. To date, there is a limited knowledge about the onset or the divergent pathways of developmental regression, due to a complex interaction between biological and environmental factors which in turn presenting many clinical challenges [3]. Children with developmental regression are characterized by neurological disabilities such as ataxia, epilepsy, vision or hearing impairment, movement disorders, and sleeping and behavioral problems [4]. Developmental regression is associated with diagnoses of childhood disintegrative disorders, Landau Kleffner syndrome, Phelan McDermid syndrome, and neurodegenerative diseases such as neuronal ceroid lipofuscinoses (NCLs) [5]. It is extremely important to make a precise diagnosis of developmental disorders to allow appropriate and specific therapies, which may change disease progression and improve quality of life in some cases. Loss of motor, language, and social skills can be treated with occupational physical and speech therapies [6]. Neurodegenerative disorders of childhood are complicated diseases, and their diagnosis signifies a great challenge to clinicians. They include a heterogeneous group of diseases that result from specific genetic and biochemical defects such as Alzheimer’s disease, Parkinson’s disease, Huntington’s disease, amyotrophic lateral sclerosis, frontotemporal dementia, and the spinocerebellar ataxias. Also, many inherited metabolic disorders with neural regression especially NCLs [7]. The clinical approach of these disorders counts on the age of onset and developmental abnormalities in the brain. Early clinical diagnosis leads to better management and accurate genetic counseling [8]. NCLs are a group of autosomal recessive hereditary lysosomal storage disorders that cause progressive neurodegenerative diseases, with an incidence of about 1–3/100.000 and a prevalence of about 2–4/1.000.000. They are associated with motor and cognitive regression, cerebellar atrophy, vision loss, ataxia, epilepsy, seizures, and a shortened lifespan. According to genetic classification, NCLs are 14 types [9]. As rare disease entities, NCLs comprise the most common cause for progressive neurodegenerative disease in children. NCLs are clinically classified into four major types based on the age of onset of the disease: infantile (6–24 months), late infantile (2–4 years), juvenile (5–10 years), and adult-onset (> 18 years) [10]. Biochemical and genetic studies especially whole exome sequencing (WES) are considered to be the main diagnostic tools of NCLs. Over the last 2 decades, knowledge of the molecular basis of NCLs has been achieved, but the precise pathomechanisms leading to cell and neuronal death have not yet been illuminated. A few targeted therapies for the NCLs, including enzyme replacement and gene therapies, have become available for human study recently, but only one product is commercially available at present for type NCL2. There is an urgent need to find safe and effective treatments for rare neurodegenerative diseases, such as the NCLs, as well as mutual agreements between families of patients and health systems together with pharmaceutical companies [11]. Due to the complexity of genetic diseases, next-generation sequencing (NGS) technology had led to great advances in understanding the causes of Mendelian and neurological diseases. To improve clinical and genetic diagnosis as well as providing significant treatments, a precise genetic test should be selected depending on the rapid quality of time, cost-effectiveness, coverage area, and sequencing range. WES is an appropriate method for finding new mutations and thousands of variants, including missense variants, protein-truncating variants, and large structural variants (SVs); however, whole genome sequencing (WGS) is suitable for exploring the roles of specific and de novo genes in neurodegenerative disorders. A combination of clinical and molecular analysis has become a more effective diagnosis approach [12]. We aimed to highlight genes causing developmental regression and neurodegenerative disorders in Egypt using NGS-based analyses, including interpretations of different variants and mutations.

## Subjects and methods

### Participants

The present study was performed through years from 2017 to 2022 on 763 Egyptian children, most of them from consanguineous families (78%) of age ranged from 1 to 11 years who were presented with seizures, ataxia, visual impairment, and developmental regression. Affected individuals were subjected to detailed clinical evaluation, family history, and specific neurological examination; an MRI brain scan was requested when needed. Participants were recruited from the Centre of Excellence of Medical Research, National Research Centre, Cairo, Egypt. An informed consent form was signed from parents or guardian that was approved by the Medical Research Ethics Committee, NRC.

### Clinical investigations

The inclusion criteria were seizures, developmental regression, myoclonus, visual failure, and ataxia. However, exclusion criteria include brain trauma and medication intake.

### Molecular analysis

DNA from blood samples was extracted from each sample of 763 participants using Thermo Scientific Gene JET Genomic DNA Purification Kit (#K0721, Thermo Scientific, Waltham, MA, USA) according to the manufacturer’s instructions. The concentration and purity of DNA were quantified using a nano-drop spectrophotometer device kit (Thermo Scientific, USA) and stored in aliquots at − 20 °C.

#### Whole exome sequencing

A total of 50 ng of genomic DNA of 763 children is fragmented to target regions using DNA capture probes. These regions include approximately 41 Mb of the human coding exome (targeting > 98% of the coding RefSeq from the human genome build GRCh37/hg19). The generated library is sequenced on an Illumina platform to obtain at least 20 × coverage depth for > 98% of the targeted bases. An in-house bioinformatics pipeline including read alignment to GRCh37/hg19 genome assembly and revised Cambridge Reference Sequence (rCRS) of the Human Mitochondrial DNA (NC_012920), variant calling, annotation, and comprehensive variant filtering is applied. All variants with minor allele frequency (MAF) of less than 1% in gnomAD database and disease-causing variants reported in HGMD®, in ClinVar, or in CentoMD® are evaluated. The investigation for relevant variants is focused on coding exons and flanking + / − 10 intronic nucleotides of genes with clear gene-phenotype evidence (based on OMIM® information). Variants with low sequencing quality and/or unclear zygosity are confirmed by orthogonal methods. Consequently, a specificity of > 99.9% for all reported variants is warranted. The copy number variation (CNV) detection software has a sensitivity of more than 95% for all homozygous/hemizygous and mitochondrial deletions, as well as heterozygous deletions/duplications and homozygous/hemizygous duplications spanning at least three consecutive exons. For the uniparental disomy (UPD) screening, a specific algorithm is used to assess the well-known clinically relevant chromosomal regions (6q24, 7, 11p15.5, 14q32, 15q11q13, 20q13, and 20). Selected variations were obtained from WES with minor allele frequencies < 0.05 using the following databases (dbSNP, 1000 Genomes Project). Effects of single nucleotide variants (SNVs) were predicted by SIFT, Polyphen-2, PROVEAN, and Mutation Taster programs. The novel mutation was confirmed using Gnomad and Novel Taster.

#### Sanger sequencing

Sanger sequencing was performed to confirm identified mutations of candidates. Polymerase chain reaction (PCR) of genomic DNA was performed in thermal cycler (Perkin-Elmer; USA) using specific primers that were designed referring to genomic sequence (GenBank accession numbers and Qiagen Taq PCR Core kit: USA). PCR primers were designed using the Primer3 program, and all sequences of the primers are available on request. PCR amplicons were purified using enzymatic Exonuclease/Shrimp Alkaline phosphatase treatment (Sigma, USA) that was held in (PerkinElmer). Relevant PCR products were covered by both forward and reverse strand sequencing using the BigDye Terminator v1.1 Cycle Sequencing Kit (Applied Biosystems, Carlsbad, CA, USA) and analyzed on the system (ABI 3130 Genetic Analyzer). Mutational analysis was carried using FinchTV 1.4.0 software.

## Results

### Participants and ethics statement

The current study comprised 763 Egyptian children (their age ranged from 1 to 11 years), were collected from years 2017 to 2022; 595 (78%) of them were offspring of consanguineous marriages. Affected individuals were subjected to clinical details including, family history, pedigree analysis, neurological examination, EEG, and MRI brain scan that was requested when needed. Patients were recruited from the Centre of Excellence of Medical Research, National Research Centre, Cairo, Egypt, and their parents or guardian signed an informed consent approved by the Medical Research Ethics Committee at NRC for the patient that was approved by the Medical Research Ethics Committee, NRC. All data were anonymous and coded to assure the confidentiality of participants.

### Clinical investigations

**Fig. 1:**
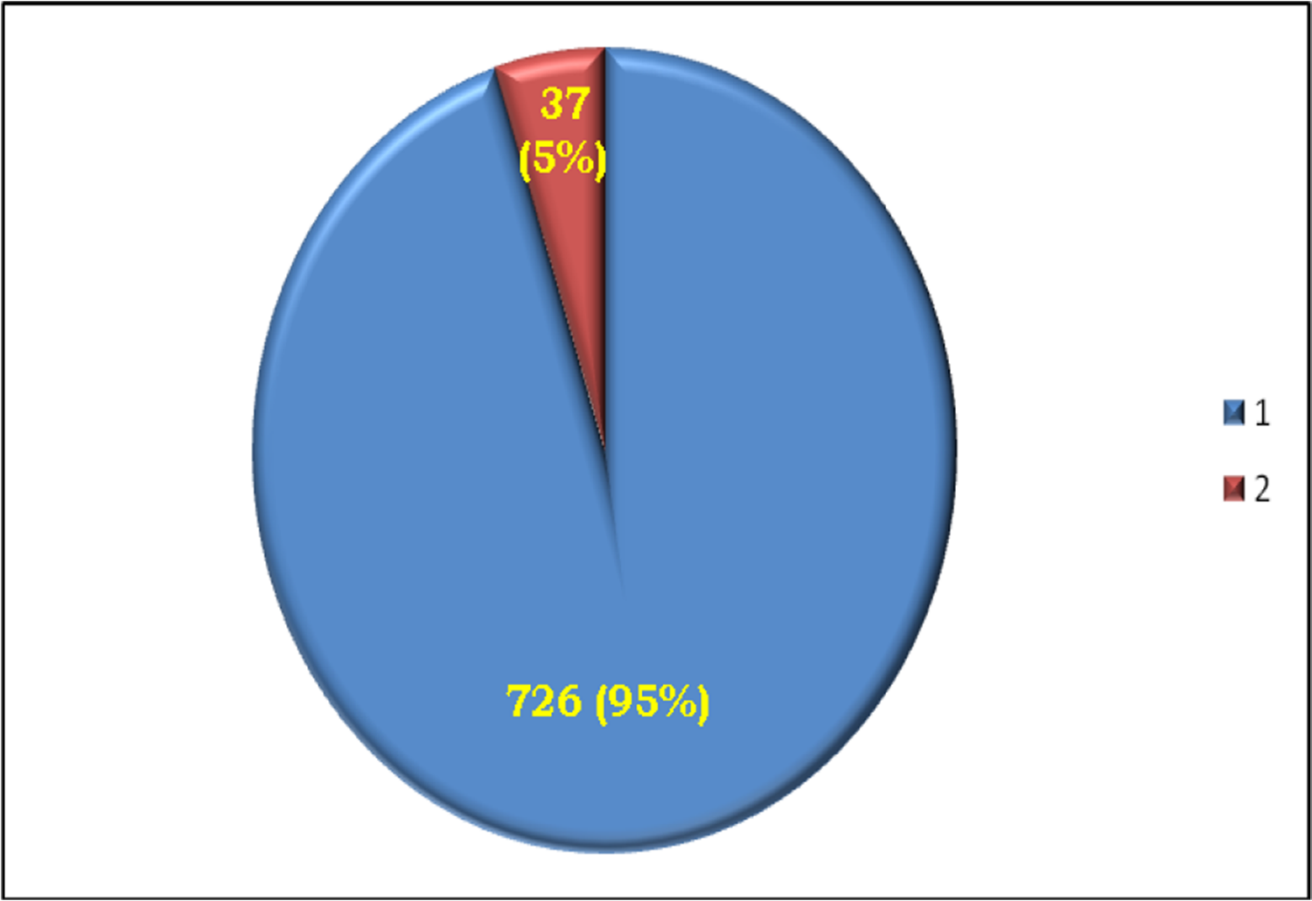
726 (95%) of 763 candidates were previously clinically and molecularly investigated; meanwhile, 37 (5%) of 763 were recently received and were suspected with NCL symptoms

**Fig. 2:**
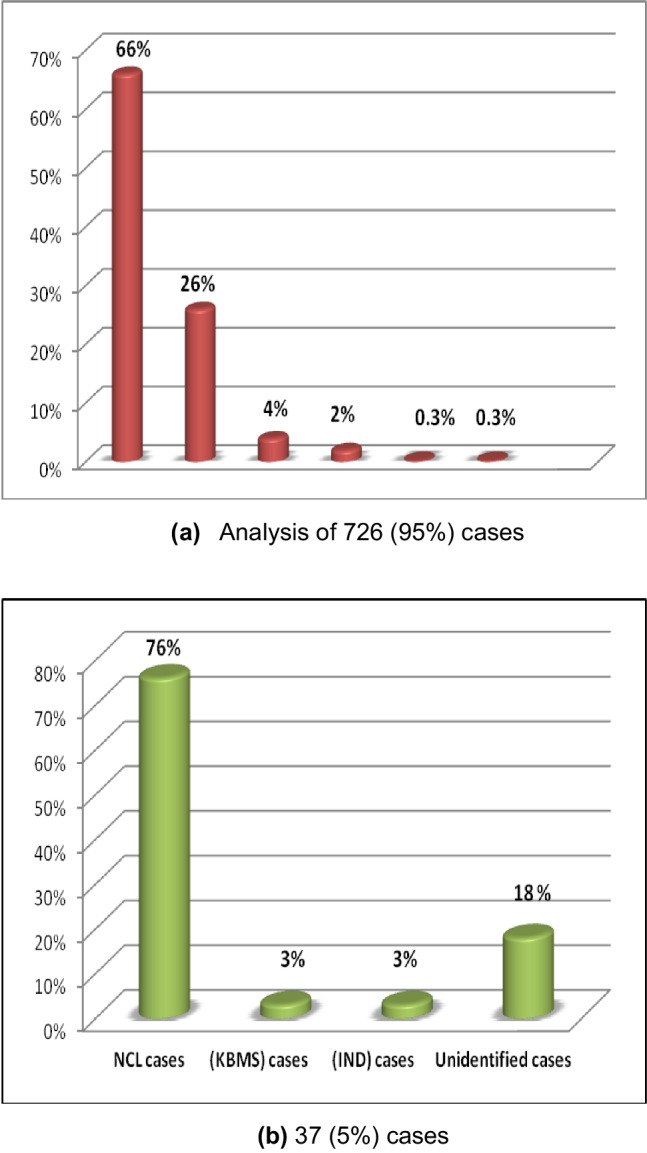
(**A)** Analysis of 726 (95%) cases; (66%) cases had pediatric stroke, congenital infection, and hepatic encephalopathy, (26%) dymorphic features, (4%) central nervous system (CNS) malformation, (2%) leukodystrophy, (0.3%) ataxia telangiectasia, and (0.3%) tuberous sclerosis. **b** Analysis of 37 (5%) cases, (76%) NCL disorders, (3%) Kaya-Barakat-Masson syndrome (KBMS) patient, (3%) infantile neuroaxonal dystrophy (IND), and (18%) unidentified cases

A total of 726 (95%) cases out of 763 Egyptian children were early clinically and molecularly analyzed. From 726 participants, 482 (66%) cases showed demonstrated hypoxic-ischemic encephalopathy, pediatric stroke, congenital infection, and metabolize encephalopathy; meanwhile, 192 (26%) had clearly dymorphic features, 31 (4%) individuals were described with central nervous system (CNS) malformation, 17 (2%) cases were illustrated with leukodystrophy, 2 (0.3%) had ataxia telangiectasia, and 2 (0.3%) cases were diagnosed with tuberous sclerosis. The remained 37 (5%) out of 763 candidates were received recently and were suspected with NCL symptoms; however, 28 (76%) cases were verified with NCL disorders, 1 (3%) patient was Kaya-Barakat-Masson syndrome (KBMS), and another 1 (3%) was diagnosed as infantile neuroaxonal dystrophy (IND). Seven (18%) out of the remained 37 candidates were undefined cases that required WGS for further molecular diagnosis (Figs. 1 and 2). All patients had abnormal EEG. However, the MRI scan showed various degrees and combination of cerebellar-cerebral atrophy. The main investigated criteria were seizures, ataxia, developmental regression, physical abnormalities, myoclonus, and visual failure (Table 1).

**Table 1:** The main clinical characteristics of enrolled patients

Patient number	Disease	Inheritance	Age (years)	Gender	Symptoms	Brain MRI	Ultrastructural findings
	CLN1 disease	AR	2	Female	Cognitive and motor decline, vision loss, hypotonia, myoclonus, seizures, ataxia, acquired microcephaly	Cerebellar atrophy	Granular osmiophilic deposits (GROD)
	CLN1 disease	AR	4	Male	Developmental regression, early cognitive decline, later vision loss, ataxia, myoclonus, seizures	Cerebral atrophy	Granular osmiophilic deposits (GROD)
	CLN1 disease	AR	3	Male	Developmental regression, seizures, speech and visual loss	Brain atrophy	Granular osmiophilic deposits (GROD)
	CLN1 disease	AR	4		Motor decline, seizures, ataxia, spasticity, later vision loss	Cerebellar atrophy	Granular osmiophilic deposits (GROD)
	CLN2 disease	AR	7	Male	Developmental regression, language, visual loss, and spasticity	Brain atrophy	Curvilinear profiles (CVPs)
	CLN2 disease	AR	6	Male	Developmental regression, ataxia, loss of acquired milestone, abnormal movement, hypotonia, and vision loss	Cerebral atrophy	Curvilinear profiles (CVPs)
	CLN2 disease	AR	5	Male	Developmental regression, speech delay, seizures	Cerebellar-cerebral atrophy	Curvilinear profiles (CVPs)
	CLN3 disease	AR	9	Female	Abnormal retinal morphology, developmental regression, seizures, macular edema, visual impairment, decline mental and motor capacities	Cerebellar atrophy	Fingerprint profiles (FPPs)
	CLN3 disease	AR	8	Female	Inability to walk, mental deterioration, motor delay, myoclonic seizure	Gray matter heterotopia	Fingerprint profiles (FPPs)
	CLN5 disease	AR	7	Male	Decline of mental and motor capacities, epilepsy, and vision loss through retinal degeneration	Brain atrophy	Rectilinear profiles (RLP)
	CLN5 disease	AR	5	Female	Behavioral changes, hyperactivity, decreased attention span, ataxia, movement impairment, myoclonic, seizures, and visual loss	Cerebellar atrophy	Fingerprint profiles (FPPs)
	CLN5 disease	AR	6	Male	Abnormality of vision, delayed speech, epileptic spasm, myoclonic jerks, motor delay, and seizure	Cerebellar atrophy	Curvilinear profiles (CVPs)
	CLN6 disease	AR	6	Male	Abnormality of vision, delayed language development, epileptic spasm, mental and motor deterioration, and ataxia	Brain atrophy	Granular osmiophilic deposits (GROD)
	CLN6 disease	AR	4	Female	Myoclonus, ataxia, delayed language, behavioral problems, visual loss, and ataxia	Cortical atrophy	Curvilinear profiles (CVPs)
	CLN6 disease	AR	3	Male	Developmental regression, tonic seizures, speech impairment, visual loss, ataxia, myoclonus, and mental deterioration	Mild cerebellar atrophy	Curvilinear profiles (CVPs)
	CLN6 disease	AR	5	Male	Behavioral problems, seizure, motor symptoms, vision, disease	Cerebral atrophy	Granular osmiophilic deposits (GROD)
	CLN7 disease	AR	6	Male	Abnormal movements, neurodevelopmental regression, seizures, and retinal dystrophy	Abnormal brain atrophy	Fingerprint profiles (FPPs)
	CLN7 disease	AR	5	Female	Progressive mental and motor deterioration, myoclonus, visual failure, and ataxia	Brain atrophy	Curvilinear profiles (CVPs)
	CLN7 disease	AR	8	Female	Psychomotor decline, movement abnormalities, ataxia, impairment of vision	Cerebral and cerebellar atrophy	Fingerprint profiles (FPPs)
	CLN7 disease	AR	5	Male	Mental and speech regression, developmental regression, hypotonia, and seizure	Cerebral white matter signal changes on the periventricullar regions	Rectilinear profiles (RLP)
	CLN7 disease	AR	8	Male	Developmental regression, delayed speech, visual loss, ataxia gait, and hypotonia	Moderate cerebral atrophy	Curvilinear profiles (CVPs)
	CLN7 disease	AR	6	Female	Seizures, visual failure motor, ataxia, regression, and mental deterioration	Cortical atrophy	Curvilinear profiles (CVPs)
	CLN8 disease	AR	11	Male	Developmental regression, delayed speech, poor eye contact, seizures, and hypotonia	Cerebellar hypoplasia	Curvilinear profiles (CVPs)
	CLN10 disease	AR	5	Male	Developmental regression, language and motor delay, visual impairment, epilepsy, and hypotonia	Brain atrophy	Granular osmiophilic deposits (GROD)
	CLN13 disease	AR	5	Female	Seizures, myoclonus, cognitive decline, depression, and visual failure	Cerebellar tremor	Fingerprint profiles (FPPs)
	CLN14 disease	AR	7	Male	Ataxia, loss of acquired milestone, abnormal movement, hypotonia, developmental regression, and vision loss	Cerebellar atrophy	Granular osmiophilic deposits (GROD)
	CLN14 disease	AR	6	Female	Epilepsy, motor delay, hypotonia, and visual failure	Cerebellar atrophy	Rectilinear profiles (RLP)
	Leukoenthephalopathy with ataxia	AR	6	Male	Developmental regression, ataxia, visual defects, headache, and delayed speech	Signal of abnormalities in gangalia	-
	Infantile neuroaxonal Dystrophy (INAD)	AR	3	Male	Psychomotor regression, ataxia, visual loss, abnormality of movement, and hypotonia	Cerebellar atrophy	-
	Kaya-Barakat-Masson syndrome (KBMS)	AR	6	Male	Developmental regression, hypotonia, spasticity, central hypoventilation, poor eye contact, absence of speech, and seizures	Brain atrophy	-

*AR* autosomal recessive

### Molecular analysis

Molecular screening using whole exome sequencing and Sanger sequencing of 37 Egyptian children revealed 30 mutations in different causative genes with autosomal recessive inheritance pattern. A total of 28 mutations have been investigated in genes (*CLN1*, *CLN2*, *CLN3*, *CLN5*, *CLN6*, *CLN7*, *CLN8*, *CLN10*, *CLN13*, *CLN14*, and *CLCN2*) in 28 NCL patients; one mutation was detected in *YIF1B* gene in one patient who showed symptoms of Kaya-Barakat-Masson syndrome, and one was demonstrated in *PLA2G6* gene of one patient who was suspected to have infantile neuroaxonal dystrophy disease. Seven cases required further molecular studies using the whole genome sequencing technique (Table 2).

**Table 2:** Classification of enrolled genes

Locus name	OMIM	Gene	Chromosome location	Phenotype
CLN1	600,432	*PPT1*	1p34.2	Infantile (INCL), late infantile (LINCL), juvenile (JNCL), adult (ANCL), (Kufs disease)
CLN2	607,998	*TPP1*	11p15.5	Late infantile (LINCL), juvenile (JNCL)
CLN3	607,042	*CLN3*	16p11.2	Juvenile (JNCL), adult (ANCL), (Kufs disease)
CLN5	608,102	*CLN5*	13q22.3	Late infantile (LINCL), adult (ANCL), (Kufs disease)
CLN6	606,725	*CLN6*	5q21-23	Late infantile (LINCL), juvenile (JNCL), adult (ANCL), (Kufs disease)
CLN7	611,124	*MFSD8*	4q28.2	Late infantile (LINCL)
CLN8	607,837	*CLN8*	8p23.3	Late infantile (LINCL), northern epilepsy (NE)
CLN10	116,840	*CTSD*	11p15.4	Congenital late infantile (LINCL), adult (ANCL), (Kufs disease)
CLN13	603,539	*CTSF*	11q13.2	Adult (ANCL), (Kufs disease)
CLN14	611,725	*KCTD7*	7q11.21	Infantile (INCL)
CLCN2	600,570	*CLCN2*	3q27. 1	Infantile
YIF1B	619,109	*YIF1B*	19q13.2	Infantile
PLA2G6	603,604	*PLA2G6*	22q13.1	Infantile

#### Sanger sequencing

In the present study, we started with applying the direct Sanger sequencing technique of *CLN6* and *CLN7* genes as the most common genes of NCLs disease, on the recent 37 Egyptian children with suspected neurodegenerative symptoms. It revealed 6 pathogenic missense mutations in 6 unrelated candidates. Two reported mutations c.406C > T (p.Arg136Cys) and c.896C > T (p.Pro299Leu) were detected in *CLN6* gene, and three mutations were described in *CLN7* gene; one of them was novel mutation c.600 G > A (p.Trp200Ser), and the other two mutations were reported c.416G > A (p.Arg139His) and c.881C > A (p.Thr294Lys). One mutation c.789G > C (p. Trp263Cys) was investigated in *CLN8* gene in patient who had symptoms and age of onset of suspected CLN8-type (Table 3).

**Table 3:** Results of Sanger sequencing of *CLN6*, *CLN7*, and *CLN8* genes

Patient number	Mutation	Amino acid	Type of mutation	Novel/reported	Gene	Disease
	c.406C > T	p.Arg136Cys	Missense	Reported	*CLN6.*Exon 4	NCL disease
	c.896C > T	p.Pro299Leu	Missense	Reported	*CLN6.* Exon 7	
	c.416G > A	p.Arg139His	Missense	Reported	*CLN7* Exon 5	
	c.600 G > A	p.Trp200Ser	Missense	Novel	*CLN7*. Exon 7	
	c.881C > A	p.Thr294Lys	Missense	Reported	*CLN7.* Exon 10	
	c.789G > C	p.Trp263Cys	Missense	Reported	*CLN8.* Exon 3	

#### Whole exome sequencing

**Fig. 3:**
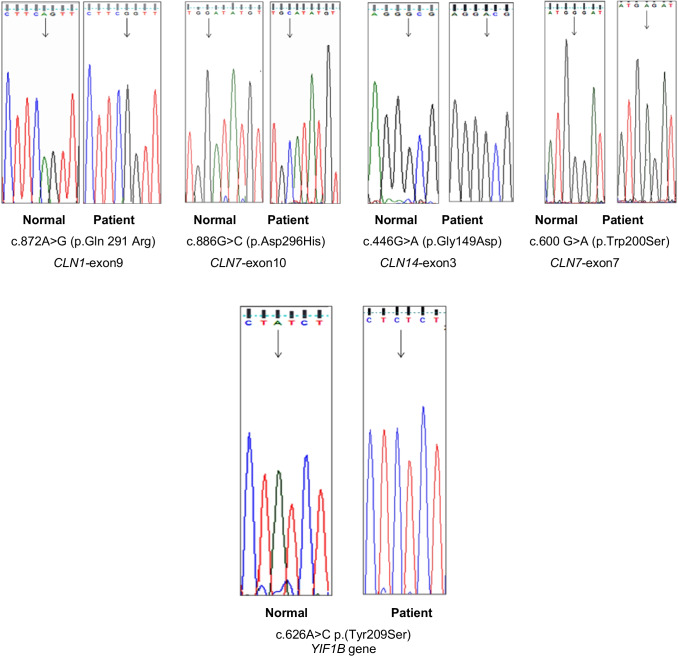
Electropherograms of novel mutations of *CLN* genes (*CLN1*, *7*, *14*) and *YIF1B* gene

Whole exome sequencing was carried out on remained 31 affected probands. No variants were detected in the genes of 7 cases which recommend further genomic sequencing investigation. Two pathogenic homozygous mutations of 2 different diseases were reported in two unrelated patients; one is a novel mutation c.626A > C (p.Tyr209Ser) in gene *YIF1B* in one patient with suspected symptoms of Kaya-Barakat-Masson syndrome, and the other one is a reported mutation c.1039G > A (p.Gly347Arg) in *PLA2G6* gene in one patient of pre-diagnosed symptoms of infantile neuroaxonal dystrophy disease. Among 22 pathogenic mutations of *CLN* genes, 4 novel homozygous missense mutations have been reported in 4 different genes: c.872A > G (p.Gln 291 Arg) in gene *CLN1*, c.886G > C (p.Asp296His) in gene *CLN7*, c.446G > A (p.Gly149Asp) in gene *CLN14*, and the fourth one (c.906 + 2 T > A) was in donor splice region of intron 11 of *CLN3* gene. The remained 18 pathogenic mutations were previously reported. They included 14 homozygous missense mutations: 2 in *CLN1* gene, 3 in *CLN2* gene, 2 in *CLN5* gene, 1 in *CLN6* gene, 2 in *CLN7* gene, 1 in *CLN10* gene, 1 in *CLN13* gene, another one in *CLN14* gene, and one in acceptor splice region c.395_396delCT (p.Ser132CysfsX18) in *CLCN2* gene, in addition to 4 deletion mutations c.644delA (p.Tyr215SerfsX5) in *CLN1* gene, c.424delG (p.Val142Leufs*39) in *CLN3* gene, c.919del (p.Arg307Glufs*29) in *CLN5* gene, and c.395_396delCT (p.Ser132CysfsX18) in *CLN6* gene (Table 4). All novel mutations were confirmed using dbSNP, 1000 Genomes Project, PROVEAN, PolyPhen2, SIFT, Mutation Taster, Gnomad, and Novel Taster. All variants detected in whole exome sequencing were confirmed together with those of their parents using the Sanger sequencing technique (Fig. 3). Both parents of probands were identified as carriers of the mutations.

**Table 4:** Results WES technique of different genes

Patient number	Mutation	Amino acid	Type of mutation	Novel/reported	Gene	Disease
	c.117 T > A	p.His39Gln	Missense	Reported	*CLN1.*Exon1	NCLs disease
	c.364A > T	p.Arg122Trp	Missense	Reported	*CLN1.* Exon 4	
	c.644delA	p.Tyr215SerfsX5	1-bp deletion	Reported	*CLN1.* Exon 7	
	c.872A > G	p.Gln 291 Arg	Missense	Novel	*CLN1.* Exon 9	
	c.229G > A	p.Gly77Arg	Missense	Reported	*CLN2*.Exon 3	
	c.457 T > C	p.Ser153Pro	Missense	Reported	*CLN2.* Exon 5	
	c.1016G > A	p.Arg339Gln	Missense	Reported	*CLN2.* Exon 8	
	c.424delG	p.Val142Leufs*39	1-bp deletion	Reported	*CLN3.* Exon 6	
	c.906 + 2 T > A	Donor splice region	Missense	Novel	*CLN3.* Intron 11	
	c.613C > T	p. Pro205Ser	Missense	Reported	*CLN5.* Exon 3	
	c.919del	p. Arg307Glufs*29	1-bp deletion	Reported	*CLN5.* Exon 4	
	c.1137G > T	p. Trp379Cys	Missense	Reported	*CLN5.* Exon 4	
	c.395_396delCT	p.Ser132CysfsX18	2-bp deletion	Reported	*CLN6.* Exon 4	
	c.485 T > G	p.Leu162Arg	Missense	Reported	*CLN6.* Exon 4	
	c.479C > T	p.Thr160Ile	Missense	Reported	*CLN7.* Exon 6	
	c.886G > C	p.Asp296His	Missense	Novel	*CLN7.* Exon 10	
	c.1235C > T	p.Pro412Leu	Missense	Reported	*CLN7*.Exon 12	
	c.1196G > A	p.Arg399His	Missense	Reported	*CLN10.* Exon 9	
	c.1439C > T	p.Ser480Leu	Missense	Reported	*CLN13.* Exon 13	
	c.446G > A	p.Gly149Asp	Missense	Novel	*CLN14* Exon *3*	
	c.550C > T	p.Arg184Cys	Missense	Reported	*CLN14.* Exon 4	
	c.1856-3C > T	Acceptor Splice region	Missense	Reported	*CLCN2*	
	c.1039G > A	p.Gly347Arg	Missense	Reported	*PLA2G6*	Infantile neuroaxonal dystrophy
	c.626A > C	p.Tyr209Ser	Missense	Novel	*YIF1B*	Kaya-Barakat-Masson syndrome

## Discussion

Developmental regression (DR) is the progressive loss of previously acquired skills after normal developmental phase especially in children. It is considered as an ominous sign for a genetic disorder, associated with neurodegenerative conditions including neuronal ceroid lipofuscinosis disease and though presenting many clinical challenges [13]. Children diagnosed with developmental regression are suffering from epilepsy, ataxia, vision loss, movement disabilities, sleeping impairment, and behavioral problems [14]. The present study enrolled 763 Egyptian children of age ranged from 1 to 11 years. They were clinically diagnosed with neurodevelopmental regression symptoms as physical disability, congenetive defects, seizures, ataxia, and visual impairment [15]. A total of 726 (95%) cases of 763 Egyptian candidates included different neurodegenerative diseases as 482 (66%) cases demonstrated stroke, congenital infection, and hepatic encephalopathy, 192 (26%) were presented with clearly dymorphic features, 31(4%) individuals were illustrated with CNS malformation, 17 (2%) showed leukodystrophy, 2 (0.3%) were pre-diagnosed with ataxia telangiectasia, and 2 (0.3%) cases had tuberous sclerosis. The remained 37 (5%) individuals were suspected with NCL symptoms (cognitive decline, speech impairment, epilepsy, ataxia, progressive visual loss, and motor deterioration; however, 28 cases were validated to be NCL patient, 1 was Kaya-Barakat-Masson syndrome, and 1 (3%) was diagnosed as infantile neuroaxonal dystrophy. The thirty-seven cases were molecularly diagnosed using the Sanger sequencing and WES [16]. Molecular analysis of 37 Egyptian children using whole exome sequencing and Sanger sequencing revealed 30 pathogenic mutations in different causative genes of neurodegenerative diseases with autosomal recessive inheritance pattern. Twenty-eight mutations have been detected in genes (*CLN1*, *CLN2*, *CLN3*, *CLN5*, *CLN6*, *CLN7*, *CLN8*, *CLN10*, *CLN13*, *CLN14*, and *CLCN2*) of 28 unrelated NCL patients [17]; one mutation was found in *YIF1B* gene in one patient with symptoms of Kaya-Barakat-Masson syndrome, and one was illustrated in *PLA2G6* gene of one patient with suspected to have infantile neuroaxonal dystrophy disease, and 7 cases required whole genome sequencing for further molecular investigation [18]. Two pathogenic homozygous mutations of 2 different neurodevelopmental diseases were reported in two unrelated patients; the first one is a novel mutation c.626A > C (p.Tyr209Ser) in gene *YIF1B* in one patient of 6 years old with suspected symptoms: developmental regression, hypotonia, spasticity, central hypoventilation, poor eye contact, absence of speech, seizures, and brain abnormalities of Kaya-Barakat-Masson syndrome. The novel mutation was confirmed using PolyPhen2, SIFT, Mutation Taster, Gnomad, and Novel Taster software [19]. The second one is a reported mutation c.1039G > A (p.Gly347Arg) in *PLA2G6* gene in one patient of age 3 years old; he was pre-diagnosed with symptoms of classical infantile neuroaxonal dystrophy [INAD] such as psychomotor regression, ataxia, visual loss, abnormality of movement, and hypotonia, and his MRI showed cerebellar atrophy. Both mutations were probably damaging with a score of 1 according to PolyPhen2 software and disease causing with a *p* value of 0.99 consistent with Mutation Taster software [20]. Pathogenic mutations in *CLN* genes (*CLN1*, *CLN2*, *CLN3*, *CLN5*, *CLN6*, *CLN7*, *CLN8*, *CLN10*, *CLN13*, *CLN14*, and *CLCN2*) represent the principle contribution among the investigated mutations, and affected patients were of age ranged from 2 to 11 years old. The main clinical features assigned to them were developmental regression, epilepsy, speech impairment, cognitive decline, vision loss, hypotonia, myoclonus, seizures, and ataxia, and their MRI scan showed cerebellar-cerebral atrophy [21]. Five novel homozygous missense mutations were reported in different genes of 5 unrelated patients; one of them c.600 G > A (p.Trp200Ser) was detected in *CLN7* gene using the Sanger sequencing technique only, and the other 4 mutations c.872A > G (p.Gln 291 Arg), c.886G > C (p.Asp296His), c.446G > A (p.Gly149Asp), and (c.906 + 2 T > A) were described in genes *CLN1*, *CLN7*, *CLN14*, and donor splice region of intron 11 of *CLN3* gene, respectively, using WES. In silico analysis was performed to predict the effect of the variants, using the Polymorphism Phenotyping v2 (PolyPhen-2) (http://genetics.bwh.harvard. edu/pph2/dbsearch. shtml), the PROVEAN (http://provean.jcvi.org/index.php), the SIFT (http://sift.jcvi.org/www/SIFT_enst_submit.html), and MutationTaster (http://www.mutationtaster.org/index.html) prediction tools. According to the previous programs, novel mutations were confirmed to have damaging effect on protein feature with score 1 and cause diseasing with score > 0.99, and novel mutations were confirmed using Gnomad and Novel Taster [10]. The remained 23 pathogenic mutations were previously reported. They included 4 deletion mutations c.644delA (p.Tyr215SerfsX5), c.424delG (p.Val142Leufs*39), c.919del (p.Arg307Glufs*29), and c.395_396delCT (p.Ser132CysfsX18) in genes *CLN1*, *CLN3*, *CLN5*, and *CLN6* genes, respectively, which result in frameshift sequencing, cause alteration in the function of each protein, and in turn, result in causing NCL disease [22–24]. Nineteen homozygous missense mutations have been illustrated in 10 different *CLN* genes: 2 in *CLN1* gene c.117 T > A (p.His39Gln) and c.364A > T (p.Arg122Trp) [22, 25], 3 in *CLN2* gene [c.229G > A (p.Gly77Arg), c.457 T > C (p.Ser153Pro), and c.1016G > A (p.Arg339Gln)] [23], 2 in *CLN5* gene [c.613C > T (p. Pro205Ser) and c.1137G > T (p. Trp379Cys)] [23, 26], 3 in *CLN6* gene [c.406C > T (p.Arg136Cys), c.896C > T (p.Pro299Leu), and c.485 T > G (p.Leu162Arg)] [23], and 4 in *CLN7* gene [c.416G > A (p.Arg139His), c.881C > A (p.Thr294Lys), c.479C > T (p.Thr160Ile), and c.1235C > T (p.Pro412Leu)] [23]. Both *CLN6* and *CLN7* genes are the most common causative genes of NCL types [27]. Various missense mutations were detected in 5 different *CLN* genes: [c.789G > C (p.Trp263Cys)] in *CLN8* gene, [c.1196G > A (p.Arg399His)] in *CLN10*, [c.1439C > T (p.Ser480Leu)] in *CLN13* gene, [c.550C > T (p.Arg184Cys)] in *CLN14* gene, and 1 in acceptor splice region [c.1856-3C > T] in *CLCN2* gene [23, 28, 29, 32]. *CLCN2* gene provides instructions for making a chloride channel (ClC-2). ClC-2 channels are embedded within the outer membrane of most cells, and their function is thought to be particularly important in nerve cells (neurons) in the brain. Mutations in the *CLCN2* gene predict to impair the stability of the protein, which reduces channel function and may contribute to intracellular chloride accumulation or neuronal hyperexcitability and results in *CLCN2*-related leukoencephalopathy [32]. All missense mutations were predicted to be deleterious and would alter protein structure and function which results causing disease [11].

## Conclusions

This study provided NGS-based approach of the genetic causes of neurodevelopmental regression diseases and focused on issues related to NGS-based analyses, including interpretations of different variants and de novo mutations of congenital genetic diseases with complex phenotypes, which in turn contributes to genetic early diagnoses and counseling of families with neurodegenerative diseases. We investigated 30 different mutations in 30 Egyptian children molecularly diagnosed with WES that mainly comprised 6 novel mutations and highlighted 3 rare neurodevelopmental diseases (Kaya-Barakat-Masson syndrome, classical infantile neuroaxonal dystrophy [INAD], and *CLCN2*-related leukoencephalopathy). We assumed that this study would be a part of demographic screening and a platform for better data communication and diagnostic experience sharing between clinicians for further investigation.
